# Editorial: Advances in research on the role of inflammation in gut functional disorders

**DOI:** 10.3389/fphys.2023.1248333

**Published:** 2023-07-07

**Authors:** Li-Fei Zheng, Sumei Liu, Jin-Xia Zhu

**Affiliations:** ^1^ Department of Physiology and Pathophysiology, School of Basic Medical Sciences, Capital Medical University, Beijing, China; ^2^ Department of Biology, College of Science and Health, University of Wisconsin-La Crosse, La Crosse, WI, United States

**Keywords:** inflammation, gut microbiome, visceral hypersensitivity, immune dysfunction, neurosecretion, gut functional disorder

## Introduction

Inflammation is often associated with the development and progression of a series of gastrointestinal disorders, such as irritable bowel syndrome (IBS), inflammatory bowel disease (IBD). Gastrointestinal inflammation can be caused by many factors, including chemical, mechanical or microbial damage, neurological, endocrine, or immune disorder. A great number of microbial species live in the gut. Although gut bacteria often drive immune activation, chronic inflammation in turn shapes the gut microbiota and contributes to dysbiosis, affecting our health and disease status. The host-microbial relationships relevant to human gastrointestinal diseases are not completely understood yet. Over the past several decades, significant progress has been made in our understanding of gastrointestinal diseases. However, its pathogenesis and etiology are far from clear. This Frontiers Research Topic reports recent findings that cover pathophysiological pathways, serum biomarker identification, and solutions to inflammation-related gut disorders.

### *Escherichia coli* challenge activating intrinsic neurosecretory reflexes in the pig colon

The enteric nervous system located within the gastrointestinal wall interacts with the immune system, gut microbiota, smooth muscle cells and epithelium to maintain mucosal barrier function and regulate motor and secretory functions (Sharkey and Mawe, 2023). Considering the physiological similarity between the porcine and the human gastrointestinal tract, the pig is suitable to be used in gastrointestinal neural mechanism study. In this Research Topic of Research Topic, Traserra et al. reported a role of *Escherichia coli* (ETEC) in activating intrinsic neurosecretory reflexes in Danbred male piglets. In their original research study, they found an increase in mast cells in the colonic mucosa and submucosa but not in the muscular layer of ETEC-infected pigs on day 9 post-challenge. ETEC infection induced increase of colonic luminal Cl^−^ secretion was mediated by activating cholinergic secretomotor neurons, the main neural component in the submucosal plexus. Besides, 5-HT released from mast cells might also be involved in activating luminal Cl^−^ secretion of animals exposed to ETEC.

### Serum autoantibody biomarker identification and massa medicata fermentata treatment for IBS

IBS is a functional bowel disorder characterized by abdominal pain or discomfort with a change in bowel habits without organic disease (Ford et al., 2020). The pathophysiology of IBS remains largely unknown. IBS is a common disorder of gut-brain interaction (Black and Ford, 2020). The diarrhea dominant IBS (IBS-D) is most common. Proposed mechanisms of IBS include visceral hypersensitivity, changes in the immune system, increased intestinal permeability, impaired gut motility, and emotional disorders. The therapeutic options for IBS include dietary, pharmacological and psychotherapeutic treatments (Lembo et al., 2022).

The diagnosis of IBS depends on symptom-based Rome criteria. However, approximately 70% of IBD patients fulfilled the IBS diagnostic criteria (Sood et al., 2016). Here, Fan et al. used the latest HuProt™ microarray method to screen clinical available serum biomarkers based on antigen-autoantibody reaction for IBS diagnosis. This study thoroughly screened autoantibodies from already known proteins of humans. They found a combination of several autoantibodies with relatively low positive rate which could differentiate IBS and healthy controls. Although some autoantibodies were related to IBS symptoms, these autoantibodies could not differentiate IBS with disease controls. This study failed to find specific autoantibodies similar to classic autoimmune diseases. It seems helpful for future studies focusing on autoimmune reaction in IBS. However, serum autoantibodies provide insufficient information for the diagnosis of IBS.

The core pathological change of IBS-D is known as “visceral hypersensitivity”. Microbial dysbiosis within the gut is thought to contribute to the pathogenesis of IBS-D, resulting in visceral sensitivity (De Palma et al., 2017). In their original research article, Zhuang et al. provided insight into the effect of Massa Medicata Fermentata (MMF), a traditional Chinese medicine, on a rat model of IBS-D with visceral hypersensitivity, established by acetic acid enema combined with restraint stress. They found that MMF treatment can reduce visceral hypersensitivity, increase the number of fecal *Bifidobacterium* and *Lactobacillus,* but decrease the amount of fecal pathogenic flagellated bacteria and Toll-like receptor 5 protein expression in the colonic mucosa in IBS-D rats. As such, they discover a potential therapeutic target for the prevention of visceral sensitivity in IBS-D.

### Overview of CD34 in IBD

IBD is a chronic, relapsing immune-mediated disease, leading to digestive disorders and inflammation (Bisgaard et al., 2022). CD34, a transmembrane glycoprotein, is expressed in mucosal dendritic cells, mast cells, eosinophils, and other immune cells (Aulakh et al., 2021). CD34 expression is abnormally increased in the intestine of IBD patients. CD34 is involved in mediating the migration of neutrophils, eosinophils, and mast cells to the inflammatory area. Its interaction with various adhesion molecules is involved in pathogenesis of IBD. Li et al. provided an overview of the role of CD34 in IBD. They reviewed the structure and localization of CD34, the effect of CD34 in recruitment and infiltration of neutrophils, migration of eosinophils, infiltration of mast cells to the inflammation sites, and the interaction between CD34 and adhesion molecules (such as selectin, integrins). They emphasized a considerable role of CD34^+^ cells in maintaining intestinal homeostasis.

### New mechanisms of lymphoid depletion in bowel obstruction

Obstructive bowel disorders (OBD) are characterized by lumen distention due to mechanical or functional bowel obstruction (BO). BO may lead to severe systemic responses, such as immune dysfunction and recurrent inflammation (Shi et al., 2018). However, the systemic responses in OBD are incompletely known. Lin et al. reported in their original study that thymus, spleen, and mesenteric lymph node (MLN) appeared atrophy in partial colon obstruction rats induced by a band in the distal colon. B and T lymphopoiesis was suppressed in the bone marrow and thymus. Depletion of gut microbiota by antibiotics blocked BO-associated lymphopenia in the MLN. Plasma levels of osteopontin (OPN) and corticosterone were increased in BO, as well as OPN expression in the distended colon. Deletion of the OPN gene attenuated suppression of lymphopoiesis in the bone marrow and thymus in BO. Their findings suggest that corticosterone and OPN in the BO colon are involved in the lymphopenia, while further study is need to confirm the role of altered gut OPN expression in lymphopenia.

## Conclusion

In summary, this Research Topic shed light on a better understanding of the role of inflammation in these related gastrointestinal diseases, provide a more details of the mechanisms underlying these disorders, and lay the foundation for novel therapies.
